# Correction: Microwave assisted green synthesis of Fe_2_O_3_/biochar for ultrasonic removal of nonsteroidal anti-inflammatory pharmaceuticals

**DOI:** 10.1039/d0ra90031a

**Published:** 2020-04-15

**Authors:** Zakaria Anfar, Mohamed Zbair, Hassan Ait Ahsaine, Amane Jada, Noureddine El Alem

**Affiliations:** Laboratoire Matériaux et Environnement LME, Faculté des Sciences, Université Ibn Zohr BP 8106, Cité Dakhla Agadir Morocco; Institute of Materials Science of Mulhouse, Haute Alsace University Mulhouse 68100 France; Laboratoire de Catalyse et Corrosion des Matériaux, Faculté des Sciences El Jadida, Université Chouaïb Doukkali BP 20 El Jadida 24000 Morocco; Chemical and Biochemical Sciences, Mohammed VI Polytechnic University Lot 660-Hay Moulay Rachid Ben Guerir Morocco hassan.aitahsaine@um6p.ma; Laboratoire de Chimie Appliqueé des Matériaux, Centre des Sciences des Matériaux Science Center, Faculty of Sciences, Mohammed V University Rabat Morocco

## Abstract

Correction for ‘Microwave assisted green synthesis of Fe_2_O_3_/biochar for ultrasonic removal of nonsteroidal anti-inflammatory pharmaceuticals’ by Zakaria Anfar *et al.*, *RSC Adv.*, 2020, **10**, 11371–11380.

In the published article there was an error in the corresponding author’s surname. The corrected version is shown here.

The Royal Society of Chemistry apologises for these errors and any consequent inconvenience to authors and readers.

## Supplementary Material

